# Adherence to the Mediterranean Diet in Paediatric Patients with Inflammatory Bowel Disease and Functional Abdominal Pain Disorders—Comparative Study

**DOI:** 10.3390/jcm14061971

**Published:** 2025-03-14

**Authors:** Iulia Florentina Ţincu, Bianca Teodora Chenescu, Larisa Alexandra Duchi, Doina Anca Pleșca

**Affiliations:** 1 Faculty of Medicine, Department of Paediatrics, “Carol Davila” University of Medicine and Pharmacy, 030167 Bucharest, Romania; iulia.tincu@umfcd.ro (I.F.Ţ.); doina.plesca@umfcd.ro (D.A.P.); 2 “Dr. Victor Gomoiu” Clinical Children’s Hospital, 030167 Bucharest, Romania; larisa.duchi@gmail.com

**Keywords:** inflammatory bowel disease, KIDMED index, functional disorders

## Abstract

**Background/Objectives:** There is a limited body of evidence regarding dietary intake in children with inflammatory bowel disease despite increasing research about the nutritional implications in the disease pathogenesis. Functional abdominal pain disorders (FAPDs) are also chronic disorders marked by chronic abdominal pain, currently described with the ROME IV criteria. This study was aimed to investigate the adherence to healthy eating habits in an inflammatory bowel disease pediatric population when compared to a matched population with functional abdominal pain gastrointestinal disorders. **Methods**: We performed a single centre study focused on dietary patterns in children with IBD and FAPDs between January 2021 and April 2024. Data collected included general information, disease phenotype, and the KIDMED index regarding healthy eating. **Results**: The final analysis was based on full data from the KIDMED index available for 122 (57 vs. 65) participants. Overall, the average KIDMED score did not vary significantly between the study population, meaning 6.89 ± 2.33 for the IBD group and 7.11 ± 2.67 for FAPDs group, *p* = 0.34. In the same KIDMED index group, mean values were higher for FAPDs patients, but results differ statistically significant only for “medium” adherence to healthy diet, showing that larger proportion of IBD patients were previously exposed to non-healthy diets: 8.99 vs. 11.1, *p* = 0.45, 5.02 vs. 6.92, *p* = 0.05, 2.89 vs. 2.56, *p* = 0.43, for group 1, 2, and 3, respectively. **Conclusions**: This study showed in our cohort that overall adherence to a healthy pattern diet is poor prior to diagnosis of different gastrointestinal pathologies in children.

## 1. Introduction

Inflammatory bowel disease (IBD) pathogenesis is highly debated, and many theories were proposed to explain this condition in children. Inflammatory bowel disease represents a global health issue with increasing incidence, including among the pediatric population. The diagnosis and management of IBD in childhood present challenges and unique characteristics due to the involvement of these conditions in crucial stages of a child’s growth and development. In recent years, the literature has increasingly emphasized the link between diet, lifestyle, and the progression of IBD [1,2]. There are a few explanations that link IBD onset and course with nutrition during childhood, supported by scientific evidence: the risk of IBD was related to some specific dietary habits and nutrients, as some dietary components may potentially be able to enhance or reduce the severity of inflammation; exclusive enteral nutrition (EEN) is first choice in some cases of pediatric Crohn’s disease, as well as variants like a Crohn’s disease exclusion diet, inducing mucosal healing without steroids. Some components excluded from the diet might influence the course of flare; malnutrition is an important marker of the disease prognosis, along with medication. In pediatric IBD, nutrition is an important target in order to assure catch-up growth and further development, bone mineral density improvement, and normal pubertal development [3]. There is a limited body of evidence regarding dietary intake in children with IBD, although there is increasing research about nutrition implications in the disease pathogenesis [4]. Most of the data focus on patients with active disease that reported either altered nutrition or follow dietary induction therapies [5].

Functional abdominal pain disorders (FAPDs), on the other hand, are also chronic disorders marked by chronic abdominal pain, with several relapsing and remitting episodes of inflammation in the gastrointestinal (GI) tract [6], currently described with the ROME IV criteria as Functional Dyspepsia, Irritable Bowel Syndrome, Abdominal Migraine and Functional Abdominal Pain Not Otherwise Specified [7]. The mechanisms implicated are still unclear, since no structural or biochemical aberrations have been found [8,9]. The major theory refers to a biopsychosocial model that involves factors like an altered gut–brain axis, altered gut motility, gut hypersensitivity, intestinal inflammation and infection, altered microbiome composition, psychological conditions, and environmental triggers, such as food and genetic predisposition [10,11].

As for diets in functional gastrointestinal disorders in general, some nutrition-based therapies improved symptoms and enhanced quality of life, although the studies are scarce, and no guideline could be promoted [12]. The science behind that is that nutrients interact with gut microbiota, modifying some phyla and species over others, improving thus intestinal barrier and intestinal motility. Concomitantly, food as a part of children’s lives, can act both as a trigger and as a cure, since some studies reported food-related symptoms in children, while excluding some nutrients from the diet ended in a diminish of clinical aspects such as pain, diarrhea, or constipation [13]. The main focus was in evaluating low fermentable oligosaccharides, disaccharides, monosaccharides, and a polyol (FODMAP) diet as a recommendation for irritable bowel syndrome in children, meaning a reduction in these nutrients, revealing overall symptom relief [14,15]. On the other hand, the Mediterranean diet (MD) is rich in FODMAP nutrients; some studies show beneficial outcomes, since adherence to the MD, in fact, can provide a reduction in inflammatory microbiota status [16,17].

The Mediterranean diet (MD) is considered to be one of the healthiest eating patterns all over the world for many age groups. It relies mainly on increased consumption of fruits, vegetables, olive oil, nuts, and cereals, with a moderate-to-high intake of fish and dairy products and a low intake of sweets, red meat, processed meats, and saturated fats [18,19]. The principal source of fat is virgin olive oil, dairy is represented by moderate amounts of cheese and yoghurt, and the diet usually requires moderate amounts of fish and red meat in low quantities [20]. One of the main benefits of this diet is that numerous studies demonstrated its role in reducing overall mortality, prevention of obesity, type 2 diabetes, cardiovascular diseases, and cancers, especially in adults [21,22]. Nevertheless, there are studies in pediatric population confirming that MD is known to reduce childhood obesity, has a role in the prevention of metabolic syndrome, as well as efficacy in attention-deficit/hyperactivity disorder and depression in children and adolescents [23,24]. There are also studies suggesting that MD could be beneficial in symptoms defining functional gastrointestinal disorders in children, due to the increased fibre and antioxidant consumption and the low intake of saturated fats and oligosaccharides [25].

In 2004, Serra-Majem et al. developed a questionnaire aimed to assess adherence to the MD among children and adolescents, particularly in the Mediterranean region and Europe [24], and many future studies have relied their practice on this objective tool. Higher adherence scores were then related to better physical activity, improved body image, and higher quality of life and sleep [26,27]. It is easy to use because of its 16 items that addresses principles sustaining Mediterranean dietary patterns and it can be both self-administered and conducted by interview. The final index ranges from 0 to 12, and it is interpreted accordingly.

This study aimed to investigate the adherence to healthy eating habits in an inflammatory bowel disease pediatric population when compared to a matched population with functional abdominal pain gastrointestinal disorders.

## 2. Materials and Methods

### Study Population

We performed a single centre prospective longitudinal study focused on dietary patterns of children diagnosed with IBD and FAPDs between January 2021 and April 2024 in the “Dr. Victor Gomoiu” Clinical Children Hospital, from Bucharest, the capital of Romania. All clinical and laboratory data, as well as baseline characteristics, were collected from medical records. We included patients aged 10–18 years old, diagnosed with IBD, according to ongoing guidelines and Porto criteria [28]. Matched subjects for age and gender were consider as control group, diagnosed with functional abdominal pain disorders Functional Dyspepsia, Irritable Bowel Syndrome, Abdominal Migraine, and Functional Abdominal Pain Not Otherwise Specified, according to ROME IV criteria [7].

**Figure 1:**
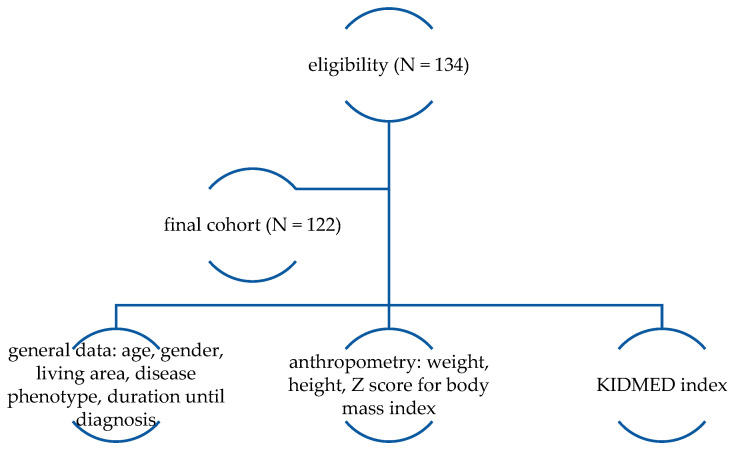
Research flowcharts.

Variables. Data collected in this study included general information: age at diagnosis (years), gender (male/female), living area (rural/urban), anthropometry assets at diagnosis (weight, height, Z score for body mass index), disease phenotype according to the Paris classification (ulcerative colitis, Crohn’s disease), and the KIDMED index regarding healthy eating. Patients with indeterminate IBD were excluded from the study. Figure 1 shows the flowchart of the investigation.

Questionnaire. The KIDMED index is based on a 16-item test that can be self-administered or conducted by interview; positive answers, proving adherence to one of the items, were noted with +1, and negative answers, meaning they were not following that item, were classified as −1; the final score consists of the sum of all value items, and it is further divided into three categories, as marked by the original author, classified as follows: (1) ≥8, optimal Mediterranean diet; (2) 4–7, improvement needed to adjust intake to Mediterranean patterns; and (3) ≤3, very low diet quality. The questionnaire was conducted at the diagnosis moment. Parents and patients were asked by the physician to answer the following items: Do they have a fruit or fruit juice every day?; do they have a second fruit every day?; do they have fresh or cooked vegetables regularly, once a day?; do they have fresh or cooked vegetables more than once a day?; do they consume fish regularly (at least 2–3 times per week)?; do they go more than once a week to a fast-food (hamburger) restaurant?; do they like pulses and eat them more than once a week?; do they consume pasta or rice almost every day (five or more times per week)?; do they have cereals or grains (bread, etc.) for breakfast?; do they consume nuts regularly (at least 2–3 times per week)?; do they use olive oil at home?; do they skip breakfast?; do they have a dairy product for breakfast (yoghurt, milk, etc.)?; do they have commercially baked goods or pastries for breakfast?; do they have two yoghurts and/or some cheese (40 g) daily?; do they have sweets and candy several times, every day? [24]. The sum of the values from the administered test was classified into three levels: (1) ≥8, optimal Mediterranean diet; (2) 4–7, improvement needed to adjust intake to Mediterranean patterns; and (3) ≤3, very low diet quality. Thus, the study population was divided into three categories, on their basis to MD adherence in order to interpret the data: group 1: scores of ≥8; group 2: scores 4–7, and group 3: scores ≤ 3, respectively. The study did not include measurement of macro and micronutrients. 

Statistics analysis. Statistical analysis was performed using a SPSS 23 software package (SPSS Inc., Chicago, IL, USA). The Chi-2 test and Mann–Whitney U test were used to compare mean values for the KIDMED index of children with IBD and children with FAPDs. A statistical significance was considered with *p* value < 0.05. The Hospital Ethics Committee approved the study (no. 9216/9 June 2023), and all caregivers signed an informed consent before adherence to the study protocol.

## 3. Results

The questionnaire was applied to 134 children, 62 with IBD and 74 with FAPDs, but the final analysis was based on full data from the KIDMED index available for 122 (57 vs. 65) participants. Overall, the average KIDMED score did not vary significantly between the study population, meaning 6.89 ± 2.33 for IBD group and 7.11 ± 2.67 for FAPDs group, *p* = 0.34. Table 1 shows the clinical characteristics of the study population, formed by 43 boys and 79 girls for the entire study population, with a mean age of 13.9 ± 2.1 years. Gender analysis showed that males scored an average of 5.88 ± 2.29 on the KIDMED and females scored an average of 7.13 ± 2.10, *p* < 0.05. No significant differences in BMI were observed across the KIDMED groups. The mean duration from the first symptoms to the final diagnosis was 4.22 ± 1.34 months and 9.66 ± 2.78 months, *p* < 0.05, for IBD and FAPDs patients, respectively.

**Figure 2:**
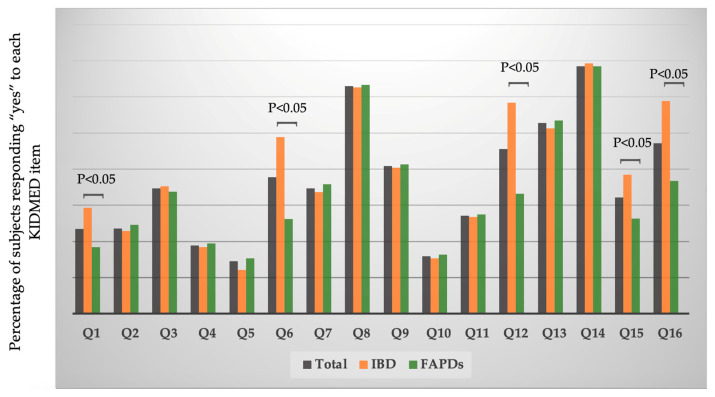
The proportion of “yes” responses for each item across the baseline diagnosis.

When analyzing the proportion of responders in the total sample with “yes” for each item of the KIDMED score, meaning marked with +1, adjusted for baseline diagnosis, we found out that Q 1 (“Has a fruit or fruit juice every day”) and Q15 (“Has two yoghurts and/or some cheese (40 g) daily”) showed a higher percentage of “yes” answers in IBD patients compared to FAPDs subjects (*p* < 0.05), meaning 29.3 vs. 18.4, and 38.5 vs. 26.3, respectively (Figure 1). Other significant differences, adjusted for baseline diagnosis and KIDMED index groups, were for questions Q6 (“Goes more than once a week to a fast-food (hamburger) restaurant”), Q12 (“Skips breakfast”), and Q16 (“Has sweets and candy several times every day”), showing that FAPDs patients responded more frequently with “no”, meaning better eating habits, the percentage being 26.2 vs. 48.9, 33.2 vs. 58.4, and 36.7 vs. 58.8, respectively (Figure 2).

**Figure 3:**
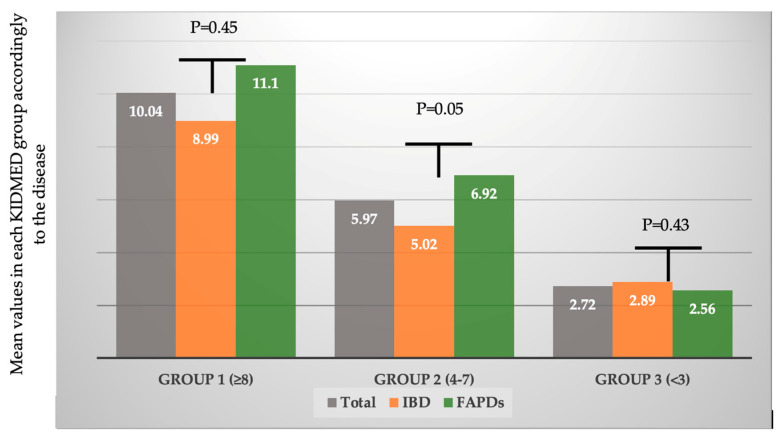
Mean values fir KIDMED index, adjusted for baseline diagnosis and groups.

In the same KIDMED index group, mean values were higher for FAPDs patients, but results differ statistically significantly only for “medium” adherence to MD, showing that larger proportion of IBD patients were previously exposed to non-healthy diets: 8.99 vs. 11.1, *p* = 0.45, 5.02 vs. 6.92, *p* = 0.05, 2.89 vs. 2.56, *p* = 0.43, for group 1, 2, and 3, respectively (Figure 3).

From the whole study population (*n* = 122), 63.93% had low adherence to the KIDMED (group 3: ≤3), 21.31% had intermediate adherence (group 2: 4–7), and 14.75% had good adherence (group 1: ≥8). Due to limited number of participants, we could not make any comparisons between IBD types of activity or FAPDS bowel habits.

## 4. Discussion

Healthy lifestyles and eating patterns during childhood will influence progression to chronic diseases later in life. The latest data suggests that the MD is beneficial for children’s long-term health and can be used in many interventional programmes for various illnesses [29]. The level of adherence to the MD is a marker of eating habits promoting health and can be assessed by the KIDMED index, shown to have a positive correlation with improved nutrient intake [26]. Unlike some other parts of Europe, and even less in some other parts of the world, in the Mediterranean countries, there is a higher knowledge about MD; even so, the adherence to healthy eating is reported to be decreasing in these areas [25].

In the present research, the authors were interested in evaluating adherence to a healthy diet by children diagnosed with IBD and FAPDs, respectively, knowing the influence of nutrition in disease pathogenesis.

It is well known that nutritional intervention is required in IBD patients, for both malnutrition recovery and induction of the remission for Crohn’s disease. The study of El Amrousy et al. showed, in a prospective trial, that MD was safe and well tolerated in pediatric and adolescent patients with active IBD and their subjects experienced clinical and laboratory improvements with normalization of inflammatory markers [30].

When analyzing the entire study population, a relatively low prevalence of high KIDMED scores is demonstrated, meaning lower adherence to MD before the diagnosis of IBD or FAPDs. Overall, only 14.75% of our subjects exhibit a high adherence to the MD and 21.31% obtained medium scores. Adherence rates found in Yu-Jin Kwon et al.’s study was up to 40–50% in Korean children [31]. Depending on geographic region, similar results are shown in Spain [32] and Italy [33]. There is a potential area of intervention in terms of promoting healthy eating habits, in general, for the pediatric population in our region since many items of preventive food intake are not followed in childhood and adolescents; this could involve communities, regulatory areas, schools, and families.

A gender repartition of the KIDMED index saw healthier adherence eating practices in girls. There are some studies focused on children and teenagers adjusted for gender motivation in terms of choosing dietary preferences; in the study of Deslippe et al. it is specifically emphasized that girls have external motivators (like eat healthier, change dietary habits around boys, and be thin to fit traditional norms) compared to boys’ motivators that are mainly internal (like gain autonomy, eat for enjoyment, and pursue gains in physical performance) [34].

Dietary practices in our country probably increased rates of obesity in our country, shown to be as high as 13.8%, 10.7%, and 5.1% for overweight, obesity, and severe obesity, respectively, in a study in the north-western part of Romania [35], highlighting the need for targeted intervention in certain population areas. Although not specific to our region, adherence to the MD could have a beneficial outcome in efforts to diminish overnutrition. As artificial intelligence techniques are becoming more and more implicated in gastrointestinal disorders [36], in terms of endoscopic techniques, there are increasing data using AI even in nutritional intervention in large populations in order to maintain adherence to healthy diets [37].

In our research, the KIDMED scores did not show statistically significant differences based on BMI; although applied previously to other populations, this is consistent to finding shown in children from Croatia [38] or from Turkey [39].

The research showed that a higher percentage of children within the IBD group receive sweets and candy several times every day; although the analysis did not include macro and micronutrient intake, this finding is similar to dietary patterns found in the same population in other studies; in Godala et al.’s study, IBD children were characterized by a high intake of total carbohydrates and their high share in dietary energy composition, with a significantly higher proportion of sucrose and a lower proportion of fibre in their diet when compared to the healthy individuals [40]. In the meantime, IBD children seem to have a higher intake of fruits and dairy products than FAPDs controls, prior to diagnosis, but those findings are slightly different from the one’s emphasized in the literature; due to the fear of symptom exacerbation, IBD populations generally reduce their intake of fruits and vegetables, which could contribute to vitamin and trace elements deficiencies, as shown in some studies [41,42]. Thus, is mandatory to add a dietician in a multidisciplinary team involved in IBD treatment.

IBD pathogenesis is linked to dysbiosis depending on the genetic background of the individuals, but changes in the gut microbiome are influenced by external factors, such as dietary intake [43]. Certain components of food intake might exert a negative effect and others could be beneficial, so there is a large interest in research about dietary modification as an environmental factor and as a preventive option for IBD [44].

We are aware of the fact that dietary differences between IBD and FAPDS are hard to interpret and are likely due to the small sample size.

Our study has several limitations that need to be expressed: from the beginning, the sample size is small and limits generalizing results and conclusions, so larger cohort data require further studies. Secondly, nutritional information achieved from well conducted nutritional assessments is missing, so any correlation regarding micronutrient association rather than eating habits cannot be analyzed. Thirdly, the singular collection data time might interfere with the solid information given by the parent, with the risk of misreporting and bias, potentially leading to overestimation or underestimation. Despite all these limitations, the research has some notable strengths, considering that, to the best of our knowledge, this is the first study addressing the adherence to the MD among Romanian children diagnosed with IBD in comparison to FAPDs children.

## 5. Conclusions

This study showed that overall adherence in the study cohort to a healthy pattern diet, meaning the Mediterranean diet, is poor prior to the diagnosis of different gastrointestinal pathologies in children, like inflammatory bowel disease or functional abdominal pain disorders. The difference between the groups in terms of dietary option did not very much, although the final diagnosis has different burdens throughout lifetime. Further preventive interventions tailored for food groups and a larger study population might promote a better adherence to dietary quality choices during childhood.

## Figures and Tables

**Table 1:** General characteristics according to KIDMED index.

	KIDMED								
	Overall		Group 1 (Score ≥ 8)		Group 2 (Score 4–7)		Group 3 (Score < 3)		*p*
	IBD	FAPDs	IBD	FAPDs	IBD	FAPDs	IBD	FAPDs	
*n*	57	65	8 (14.03)	10 (15.38)	12 (21.05)	14 (21.53)	37 (64.91)	41 (63.07)	0.05
Sex, *n* (%)									0.03
Male, *n* (%)	20 (35.09)	23 (35.38)	2 (3.58)	3 (4.61)	2 (3.58)	4 (6.15)	16 (28.07)	16 (24.61)	NS
Female, *n* (%)	37 (64.91)	42 (64.62)	6 (10.52)	7 (10.76)	10 (17.54)	10 (15.38)	21 (36.84)	25 (38.46)	NS
Age, mean ± SD (years)	13.1 ± 2.2	14.1 ± 2.6	12.5 ± 2.1	14.9 ± 3.3	13.0 ± 3.2	13.8 ± 2.8	10.1 ± 2.4	14.9 ± 3.5	NS
Height, cm	138.8 ± 16.2	144.4 ± 17.3	136.7 ± 16.1	145.2 ± 16.5	138.2 ± 16.7	143.8 ± 16.3	137.5 ± 15.4	144.6 ± 17.4	NS
Body weight, kg	35.4 ± 14.2	38.6 ± 15.3	36.5 ± 14.1	38.1 ± 15.3	34.4 ± 16.1	37.7 ± 12.3	35.1 ± 13.1	38.4 ± 15.2	NS
Z score BMI mean ± SD	−2.19 ± 0.3	1.89 ± 0.5	−1.89 ± 0.7	1.03 ± 0.3	−2.08 ± 0.8	1.67 ± 0.5	−2.21 ± 0.9	1.93 ± 0.9	NS

BMI—body mass index; NS—not significant; SD—standard deviation.

## Data Availability

The original contributions presented in this study are included in the article. Further inquiries can be directed to the corresponding author(s).

## Abbreviations

IBD	Inflammatory bowel disorder
FAPDs	Functional abdominal pain disorders
MD	Mediterranean diet
GI	Gastrointestinal
